# Surgical outcomes of patients after treatment of ruptured anterior communicating artery aneurysms: “real-world” evidence from southern Thailand

**DOI:** 10.1186/s41016-021-00259-9

**Published:** 2021-10-02

**Authors:** Kanisorn Sungkaro, Thara Tunthanathip, Chin Taweesomboonyat, Anukoon Kaewborisutsakul

**Affiliations:** Neurological Surgery Unit, Department of Surgery, Faculty of Medicine, Prince of Songkla University, 15 Karnjanavanit Street, Kho Hong, Hat Yai District, Songkhla, 90110 Thailand

**Keywords:** Anterior cerebral artery, Aneurysm clipping, Low- to middle-income countries, Neurosurgery, Subarachnoid hemorrhage

## Abstract

**Background:**

Anterior communicating artery (AComA) aneurysm rupture is the most common cause of subarachnoid hemorrhage worldwide. In this study, we aimed to determine the factors associated with a poor clinical outcome in patients with ruptured AComA aneurysms undergoing microsurgical clipping.

**Methods:**

We retrospectively reviewed the clinical and radiologic features as well as clinical outcomes of 150 consecutive patients with ruptured AComA aneurysm who underwent surgical clipping during the 11-year study period. Logistic regression analysis was performed to identify independent factors associated with unfavorable clinical outcomes (defined as a modified Rankin scale score of 3–6).

**Results:**

The study included 83 male and 67 female patients, with a mean age of 51.3 ± 11.5 years. At admission, most of the patients had good neurological status, including 97 (64.7%) patients with a Hunt and Hess grade of 1 or 2 and 109 (72.6%) patients with a World Federation of Neurosurgical Societies grade of 1 or 2. Unfavorable outcomes at 6 months were observed in 23 (22.0%) patients, and the 6-month mortality rate was 8.0%. Multivariate analysis showed that preoperative intraventricular hemorrhage (odds ratio [OR], 19.66; 95% confidence interval [CI], 5.10–75.80; *P* < 0.001), A1 hypoplasia (OR, 8.90; 95% CI, 2.82–28.04; *P* < 0.001), and postoperative cerebral infarction (OR, 3.21; 95% CI, 1.16–8.88; *P* = 0.025) were strong independent risk factors for unfavorable outcomes.

**Conclusions:**

Proper management of preoperative intraventricular hemorrhage, A1 hypoplasia, and intensive care for postoperative brain infarction are warranted for improved surgical outcomes in patients with ruptured AComA aneurysm undergoing surgical clipping.

## Background

The anterior communicating artery (AComA) is the most common location for intracranial aneurysms [1–4]. In recent years, ruptures of AComA aneurysms have been treated using endovascular treatment techniques. Nevertheless, microsurgical clipping remains the mainstay of treatment for aneurysms in this area because ruptures recur at a low rate and few subsequent treatments are needed following clipping [5].

According to recent studies, the postsurgical mortality rate for ruptured AComA aneurysms has dramatically decreased. However, morbidity rates are still high [6, 7], primarily due to the complexity and anatomical variations of blood vessels at the surgical site. Advanced surgical strategies, such as the skull-base technique, revascularization, or a combination of these along with endovascular treatments, might enable us to overcome these complexities [8]. Unfortunately, those treatments cannot always be performed, particularly in low- to middle-income countries, because they require sophisticated medical resources.

Since 1984, Songklanagarind Hospital, a tertiary-care hospital in southern Thailand, has treated patients with intracranial aneurysms who have been referred from hospitals in 14 provinces [4]. In the majority of the AComA surgeries performed at this hospital, pterional craniotomy is performed with simple direct clipping techniques. Therefore, the procedures described in this report represent the surgical management in most cases.

The aim of this study was to evaluate the clinical outcomes of patients with ruptured AComA aneurysms who underwent microsurgical clipping and the prognostic factors related to poor outcomes at our institute.

## Methods

### Patient population

This retrospective study focused on consecutive cases between January 2007 and December 2017 identified from the neurosurgical databases at our institution. The institutional ethics committee approved the protocol for this study.

We included data from all patients aged ≥ 15 years with a ruptured AComA aneurysm who underwent microsurgical clipping. Patients with incomplete preoperative vascular imaging data and unavailable medical records were excluded, as were patients with fusiform aneurysms and vascular malformations.

### Data collection

Patient characteristics, such as age, sex, presence of underlying disease, smoking, Hunt and Hess grade, and World Federation of Neurosurgical Societies (WFNS) grade at admission, were documented from the database. The patient’s preoperative status was determined using the modified Rankin scale (mRS) [9].

Preoperative imaging was reviewed by two of the authors (KS and AK). The modified Fisher scale [10] was used to classify the extent of intracranial bleeding. Vascular and aneurysmal structures were evaluated using computed tomography angiography or digital subtraction angiography, or both, depending on available data. We identified the origin and size (dome and neck width) of each aneurysm. The aneurysm dome projection was oriented in the sagittal plane, from which it could be projected predominantly into the superior, anterior, inferior, or posterior direction, according to the study by Yasargil [11].

Variations in the structure of the anterior cerebral artery (ACA), such as hypoplasia of the A1 segment, fenestrated AComA, and accessory (triplet) A2 segment, were reviewed. A1 segment hypoplasia was considered present when the A1 width on one side was < 50% of that on the contralateral side [12].

The operative complications were reviewed, and intraoperative aneurysm ruptures were documented based on the medical records. Postoperative cerebral infarction was determined to have occurred if follow-up computed tomography showed a new area of hypoattenuation [13]. Postoperative neurological deterioration was defined as a postoperative Glasgow Coma Scale score that was ≥ 2 points lower than the preoperative Glasgow Coma Scale score and persisted for > 24 h [14]. Symptomatic vasospasm was defined as a neurological deficit that reflected the function of the vascular territory for which computed tomography did not demonstrate new infarction.

The primary outcome was mortality rate during hospitalization. The secondary outcomes were neurological outcome at 6 months after the onset of aneurysmatic subarachnoid hemorrhage (aSAH), which was determined using the mRS, and factors associated with neurological outcome. Favorable and unfavorable outcomes were represented by mRS scores of 0–2 and 3–6, respectively.

### Statistical analysis

The patients characteristics and neurological outcomes were calculated as proportions for categorical and as means and standard deviations for continuous data. We performed analysis of variance to identify the prognostic factors of the 6-month neurological outcome using the mRS scores as binary variables (favorable vs. unfavorable outcome). Variables with a *P* value of < 0.1 in the univariate analysis were included in the multivariate logistic regression model. The model was selected using the backward stepwise (likelihood ratio) method. Prognostic factors with a *P* value of ≤ 0.05 were considered to indicate statistical significance.

## Results

### Patients and aneurysm characteristics

We performed microsurgical clipping in 420 patients with ruptured intracranial aneurysms, of which 150 (35.7%) had ruptured AComA aneurysms. The patients were predominantly male (83 men and 67 women) and with a mean age of 51.33 years (standard deviation, 11.49 years). Hypertension was the most common comorbidity (*n* = 45, 30.0%), followed by diabetes mellitus (*n* = 13, 8.7%). Most of the patients (*n* = 107, 71.3%) presented with a sudden headache, and most patients had excellent admission neurological status with a Hunt and Hess grade of 1 or 2 (*n* = 97, 64.7%) and a WFNS grade of 1 or 2 (*n* = 109, 72.6%). The patients’ characteristics are summarized in Table 1.

**Table 1:** Baseline patient characteristics before aneurysm clipping (*n* = 150)

Variable	***n*** (%)
Sex	
Male	83 (55.3)
Female	67 (44.7)
Mean age ± SD (years)	51.3 ± 11.5
Age	
< 60	115 (76.7)
≥ 60	35 (23.3)
Underlying disease(s)	
Hypertension	45 (30.0)
Dyslipidemia	13 (8.7)
Diabetes	4 (2.7)
Coagulopathy	4 (2.7)
Smoking	21 (14.0)
Clinical presentation(s)	
Headache	107 (71.3)
Alteration of consciousness	81 (54.0)
Vomit	60 (40.0)
Seizure	27 (18.0)
Hemiparesis	14 (9.3)
Hunt and Hess grade	
1	8 (5.3)
2	97 (64.7)
3	6 (4.0)
4	26 (17.3)
5	13 (8.7)
WFNS grade	
1	89 (59.3)
2	20 (13.3)
3	1 (0.7)
4	24 (16.0)
5	16 (10.7)
Deterioration before surgery	21 (14.0)

SD, standard deviation; WFNS, World Federation Neurological Society

Radiographic findings included isolated thin or thick aneurysmatic SAH (modified Fisher scale score, 1 and 3) in 68 (45.3%) patients. In the remaining patients (*n* = 82, 54.7%), some degree of intraventricular hemorrhage was evident. Intracerebral hemorrhage mainly within the gyrus rectus was found in 47 (31.3%) patients (Table 2).

**Table 2:** Preoperative imaging of anterior communicating artery aneurysm and significant radiographic finding (*n* = 150)

Variable	***n*** (%)
Imaging modality^a^	
CTA	4 (2.7)
DSA	134 (89.3)
Both CTA and DSA	12 (8.0)
Mean aneurysm size ± SD (mm)	
Dome size	5.47 ± 2.54
Neck size	2.96 ± 1.16
DN ratio	2.00 ± 0.98
Origin of aneurysm	
Right A1-A2	47 (31.3)
Left A1-A2	95 (63.3)
ACom	8 (5.3)
Aneurysm dome projection in sagittal plane	
Superior	54 (36.0)
Anterior	70 (46.7)
Posterior	5 (3.3)
Inferior	21 (14.0)
Multiple aneurysms	12 (8.0)
Anatomical variations	
A1 hypoplasia	81 (54.0)
Fenestrated ACom	5 (3.3)
Triplicate A2	2 (1.3)
Angiographic vasospasm	
Diffused	20 (13.3)
Local	50 (33.3)
Modified Fisher grade	
I	39 (26.0)
II	26 (17.3)
III	29 (19.3)
IV	56 (37.3)
Intracerebral hemorrhage	47 (31.3)
Intraventricular hemorrhage	82 (54.7)
Hydrocephalus	19 (12.7)

^a^Cranial computed tomography scans were included in all cases

ACom, anterior communicating artery; A1, A1 segment of anterior cerebral artery; A2, A2 segment of anterior cerebral artery; CTA, computed tomography angiography; DN, dome-to-neck; DSA, digital subtraction angiography; SD, standard deviation

Digital subtraction angiography was performed for 146 patients, and the remaining 4 patients were evaluated using computed tomography angiography for preoperative planning. Angiography revealed that the AComA aneurysms primarily originated from the left A1–A2 junction (95 patients; 63.3%) and less often from the right A1–A2 junction (47 patients; 31.3%), and true AComA aneurysms were found only in 8 (5.3%) patients. The mean width of the aneurysm neck was 2.96 ± 1.16 mm, the mean dome width was 5.47 ± 2.54 mm, and the mean dome-to-neck ratio was 2.00 ± 0.98. The aneurysm dome was projected in the anterior direction in 70 (46.7%) patients, in the superior direction in 54 (36.0%), in the inferior direction in 21 (14.0%), and in the posterior direction in 5 (3.3%). Twelve (8.0%) patients had multiple other aneurysms, none of which showed any evidence of rupture during radiographic examination (Table 2).

Variations in the structure of the ACA were detected in 85 (56.7%) patients. A1 segment hypoplasia was the most common normal variation (*n* = 81, 54.0%). In these patients, A1 segment hypoplasia was more common on the right side, and most of the aneurysms originated from the side without hypoplasia. Other variations were fenestrated AComA (*n* = 5, 3.3%) and triplicated A2 segment (*n* = 2, 1.3%) (Table 2).

### Surgery and complications

At our institution, the operation was performed as soon as possible after onset of symptoms of aneurysmatic SAH. Most of the patients (*n* = 101; 67.3%) underwent surgery within 72 h after onset. Delay was associated with delay in referral and hemodynamic instability. Twenty-one (14%) patients exhibited preoperative neurological deterioration, which was suspected to result from aneurysmal rebleeding.

The mean operative time was 339 ± 68 min, and the mean estimated blood loss was 632 ± 314 mL. Intraoperative aneurysmal rupture was observed in 22 (14.7%) patients. Surgical obliteration of the aneurysmal neck was recorded as completed obliteration (*n* = 146, 97.3%), and incomplete obliteration was achieved with muscle wrapping (*n* = 1, 0.7%). Data on obliteration was unavailable for 3 (2%) patients (Table 3).

**Table 3:** Microsurgical clipping for anterior cerebral artery aneurysm and postoperative complications (*n* = 150)

Variable	***n*** (%)
Time from onset to surgery (hours)	
< 72	101 (67.3)
≥ 72	49 (32.7)
Surgical side	
Right	51 (34.0)
Left	99 (66.0)
Aneurysm obliteration^a^	
Complete	146 (97.3)
Incomplete	1 (0.7)
NA	3 (2.0)
Interoperative rupture	22 (14.7)
Number clip(s)	
1	106 (70.6)
2	39 (26.0)
3	5 (3.3)
Mean operation time ± SD (min)	339.2 ± 68.2
Mean estimate blood loss ± SD (mL)	632.4 ± 314.6
Intracranial complications	
Cerebral infarction	55 (36.7)
Neurological worsening	38 (25.3)
Symptomatic vasospasm	33 (22.0)
Hydrocephalus	9 (6.0)
Seizure	8 (5.3)
Intracerebral hemorrhage	4 (2.7)
Systemic complications	
Acute DVT	2 (1.3)
Acute PE	1 (0.7)
Infectious complications	38 (25.3)
Pneumonia	17 (11.3)
Urinary tract infection	8 (5.3)
Meningitis	6 (4.0)
Wound infection	1 (0.7)
Mean LOS ± SD (days)	22.8 ± 38.7 (range 1–315 days)

^a^Aneurysm obliteration was recorded by neurosurgeon that might be defined by direct observation or using intravenous indocyanine green injection

DVT, deep vein thrombosis of leg; LOS, length of hospital stays; NA, not available; PE, pulmonary embolism; SD, standard deviation; UTI, urinary tract infection

Postoperative complications were divided into operation-related and systemic events. Postoperative cerebral infarction was seen in 55 (36.7%) patients, neurological deterioration in 38 (25.3%), and symptomatic vasospasm in 33 (22.0%). Eight (5.3%) patients developed seizures after surgery, but these could be controlled with a single antiepileptic drug. Postoperative infection was found in 38 (25.3%) patients, which manifested as pneumonia in 17 (11.3%) patients. Venous thromboembolism was found in 3 (2%) patients.

### Neurological outcomes

Seven (4.7%) patients died related to intracranial causes during hospitalization. The other patients were monitored at the outpatient clinic during the rehabilitation period. At postoperative 6 months, 9 (6%) patients were lost to follow-up, and 108 (71.9%) patients achieved favorable outcomes with mRS scores of 0–2. There were five out-of-hospital deaths, including three patients who died due to acute coronary disease, one patient died due to putaminal hemorrhage and one patient in whom the cause of death could not be identified. Therefore, there were a total of 12 (8.0%) deaths in the study period (Table 4).

**Table 4:** Neurological outcome in ruptured anterior communicating artery aneurysm (*n* = 150)

Variable	***n*** (%)
In-hospital mortality	7 (4.7)
mRS 6 months	
0	2 (1.3)
1	29 (19.3)
2	77 (51.4)
3	10 (6.7)
4	5 (3.3)
5	6 (4.0)
6 (deaths)	12 (8.0)
NA	9 (6.0)

mRS, modified Rankin scale; NA, not available

### Risk factors for unfavorable outcomes at 6 months

Thirty-three patients (22%) had an unfavorable outcome at postoperative 6 months. Univariate analysis showed that age ≥ 60 years, the presence of hypertension, alteration of consciousness during aneurysmatic SAH onset, poor Hunt and Hess and WFNS grades (3–5) on admission, dome-to-neck ratio < 2, A1 segment hypoplasia, preoperative intraventricular hemorrhage (IVH), postoperative cerebral infarction, and infectious complications were associated with unfavorable outcomes.

Moreover, multivariate logistic regression analysis revealed that the prognostic factors significantly associated with unfavorable outcomes were preoperative IVH (odds ratio, 19.7; 95% confidence interval, 5.1–75.8; *P* < 0.001), A1 segment hypoplasia (odds ratio, 8.9; 95% confidence interval, 2.8–28.0; *P* < 0.001), and postoperative cerebral infarction (odds ratio, 3.2; 95% confidence interval, 1.2–8.9; *P* = 0.025; Table 5).

**Table 5:** Factor associated unfavorable outcome (mRS 3-6) at postoperative 6 months in patients with ruptured anterior communicating artery aneurysm by binary logistic regression analysis (*n* = 141)

	Univariable analysis		Multivariable analysis^**a**^	
Factor	Odds ratio (95% CI)	***P*** value	Odds ratio (95% CI)	***P*** value
Female	1.49 (0.68–3.25)	0.320		
Age ≥ 60	4.17 (1.78–9.77)	0.001		
Hypertension	3.35 (1.49–7.55)	0.004		
Presenting symptom				
Headache	0.67 (0.30–1.54)	0.360		
Vomiting	1.31 (0.60–2.88)	0.500		
Alteration of consciousness	4.15 (1.66–10.38)	0.002		
Seizure	0.54 (0.17–1.70)	0.290		
Hemiparesis	2.58 (0.76–8.74)	0.130		
Deterioration prior surgery	2.56 (0.95–6.94)	0.065		
HH grades 3–5	2.38 (1.06–5.35)	0.036		
WFNS grades 3–5	2.45 (1.08–5.57)	0.033		
Aneurysm characteristics				
Neck width ≥ 4 mm	0.48 (0.15–1.51)	0.210		
DN ratio ≥ 2	0.43 (0.20–0.96)	0.039		
A1 hypoplasia	7.00 (2.51–19.50)	< 0.001	8.90 (2.82–28.04)	< 0.001
Multiple aneurysm	3.04 (0.86–10.68)	0.084		
Pre ICH	1.03 (0.44–2.42)	0.940		
Pre HCP	2.97 (0.53–16.49)	0.220		
Pre IVH	15.71 (4.51–54.75)	< 0.001	19.66 (5.10–75.80)	< 0.001
Delayed surgery	1.14 (0.50–2.61)	0.760		
Multiple clips (≥ 2)	1.71 (0.75–3.90)	0.201		
Intraoperative rupture	0.96 (0.32–2.84)	0.940		
Operative time ≥ 5 h	1.06 (0.41–2.74)	0.900		
Estimated blood loss ≥ 600 mL	1.39 (0.61–3.13)	0.430		
Complications				
Intracerebral hemorrhage	10.30 (1.03–102.67)	0.047		
Neurological worse	2.28 (0.99–5.23)	0.053		
Symptomatic vasospasm	2.34 (0.97–5.63)	0.057		
Cerebral infarction	2.96 (1.33–6.62)	0.008	3.21 (1.16–8.88)	0.025
Infectious complications	5.28 (2.28–12.23)	< 0.001		

^a^Value shows only the significant values in final model of multivariable logistic regression with backward elimination (likelihood ratio) method

CI, confidence interval; DN, dome-to-neck; HH, Hunt and Hess grade; mRS, modified Rankin scale; Pre ICH, preoperative intracerebral hemorrhage; Pre IVH, preoperative intraventricular hemorrhage; WFNS, World Federation Neurological Society

## Discussion

In this study, we evaluated the prognostic factors for unfavorable clinical outcomes after surgical clipping of ruptured AComA aneurysms. The clinical outcomes at postoperative 6 months were poor in 22% of the patients, a rate comparable with those of previous studies (range, 12.78–32.80%) [13, 15–17]. Preoperative IVH, A1 segment hypoplasia, and postoperative cerebral infarction were significant prognostic factors for unfavorable outcomes at 6 months.

IVH frequently occurs with spontaneous SAH. In the present study, more than 50% of the patients were had IVH and 19 (12.7%) patients had hydrocephalus at the onset of SAH. Rosen et al. found that 45% of patients had IVH at the onset of SAH [18]. The clinical condition of these patients tended to be poorer than that of patients without IVH. These patients tended to have more premorbid medical conditions. Furthermore, they were more likely to have thick SAH, develop vasospasm and hydrocephalus, and have poorer outcomes. Smith et al. [19] identified that the Fisher grade was correlated with symptomatic vasospasm in 50% of the patients in their study. More than 50% of the patients with large IVHs were admitted to hospitals with poor clinical grades, and the overall mortality rate was 64% [20]. IVH is a definitive independent risk factor for death, disability, and chronic hydrocephalus after SAH [21, 22].

Patients with IVH with concomitant acute hydrocephalus have markedly increased intracranial pressure and require ventricular drainage [23]. Intraventricular thrombolysis with recombinant tissue plasminogen activator, performed via ventriculostomy tube, seems to benefit patients with spontaneous IVH as it helps to normalize intracranial pressure, reduce ventricular obstruction, improve neurological function, and reduce mortality [24, 25]. The CLEAR III trial, however, did not show improved functional outcome, but it confirmed a reduction in the mortality rate [26]. Thus, IVH caused by rupture of an intracranial aneurysm is of great concern, and appropriate treatment can improve the functional outcome. Proper management of aneurysmal IVH should be clarified in future clinical trials.

A1 hypoplasia of the ACA was the most frequent anatomic variation in our study. Previous studies had documented a prevalence of A1 segment hypoplasia ranging from 24–85% in patients with AComA aneurysms [27–30]. In our study, 81 (54.0%) patients had A1 segment hypoplasia, and this is consistent with those in previous studies. The relationship between aneurysm formation and A1 segment hypoplasia may be attributed to asymmetric local inflow with chronic hemodynamic stress on the wall of the junction between the A1 and A2 segments. Moreover, the possible role of A1 segment hypoplasia in the treatment success of AComA aneurysm has also been debated. Yang et al. [30] found that A1 segment hypoplasia was related to unfavorable clinical outcomes. However, Jabbarli et al. [31], who investigated patients undergoing surgical clipping, found that A1 segment hypoplasia had no independent predictive value for functional outcome. In our study, A1 segment hypoplasia was found to be a strong independent risk factor for an unfavorable clinical outcome (*P* < 0.001). The operative risk for patients with A1 segment hypoplasia was also higher than that in patients without this anatomic variant. From our findings, we hypothesized that unfavorable outcomes result from injury to the A1 segment of ACA or that some degree of induced vasospasm is caused by temporary parent artery clipping. Patients with A1 segment hypoplasia were more likely to have ischemia in the vicinity of the ACA or infarction because no compensatory blood could reach the distal brain. In patients without A1 segment hypoplasia, compensatory blood flow from the contralateral ACA or AComA may confer a better clinical outcome. Therefore, for patients with ruptured AComA aneurysms and A1 segment hypoplasia, we strongly recommended the use of intraoperative neurophysiologic monitoring that can enable early detection of cerebral ischemia. However, if this technology is not available, particularly in situations with limited resources, temporary clipping of the parent artery should be only minimal or altogether avoided. Unfortunately, the optimal time for proximal control with temporary clipping was not established in our study.

Cerebral infarction is considered a severe complication of SAH leading morbidity and mortality. The causes of postoperative cerebral infarction are diverse. However, identifying the cause is crucial for determining a preventive strategy. A strong association of higher Fisher grades and development of cerebral vasospasm has been demonstrated [32]. As mentioned previously, Smith et al. [19] found that Fisher grade was correlated with symptomatic vasospasm in 50% of their patients; however, we found that the modified Fisher grade was not a significant factor for unfavorable clinical outcomes. Unfortunately, the power of this study, statistically, was not sufficient to further evaluate the factors related to cerebral infarction.

### Study limitations

Our study had several limitations. A major limitation was the possibility of retrospective bias. Many important factors, such as duration of temporary clipping of the A1 segment or details of intraoperative aneurysm rupture and its management, could not be defined. Second, this study included data from a single center, and sampling and selection for clipping or coiling may have created unavoidable bias. Third, this study spanned a large time period; therefore, changes in the experience of surgeons and intensive care treatments were unavoidable. Fourth, the sample size was too small to evaluate the direct effect of each significant prognostic factor. Therefore, a multicenter study should be established. However, our review of the literature revealed that no study has reported real-world results; therefore, the findings of this study should be of use for the treatment of these patients.

Finally, long-term follow-up after aneurysm treatment was not performed. Many studies have shown that the clinical outcome improves over time [33, 34]; thus, a short follow-up time might not reflect long-term outcomes. We evaluated patient outcomes over a 6-month period in this study because it was sufficient time to evaluate the short-term outcomes and because the number of patients lost to long-term follow-up could have invalidated our findings.

## Conclusions

The majority of patients with ruptured AComA aneurysms who underwent surgical clipping at our institution achieved a favorable 6-month outcome. Factors related to poor prognosis included individual anatomic conditions (A1 segment hypoplasia), disease severity (preoperative IVH), and postoperative consequence (cerebral ischemia). Further studies are warranted to evaluate and establish proper preventive strategies for better management of such patients.

## Data Availability

The datasets used in the present study are available from the corresponding author on reasonable request.

## Abbreviations

ACom: Anterior communicating artery
AComA: Anterior communicating artery aneurysm
CT: Computed tomography
CTA: Computed tomography angiography
DSA: Digital subtraction angiography
aSAH: Aneurysmal subarachnoid hemorrhage
